# Age and *APOE* genotype affect the relationship between objectively measured physical activity and power in the alpha band, a marker of brain disease

**DOI:** 10.1186/s13195-020-00681-8

**Published:** 2020-09-22

**Authors:** Jaisalmer de Frutos-Lucas, Pablo Cuesta, Federico Ramírez-Toraño, Alberto Nebreda, Esther Cuadrado-Soto, África Peral-Suárez, David Lopez-Sanz, Ricardo Bruña, Silvia Marcos-de Pedro, María Luisa Delgado-Losada, Ana María López-Sobaler, Inmaculada Concepción Rodríguez-Rojo, Ana Barabash, Juan Manuel Serrano Rodriguez, Simon M. Laws, Alberto Marcos Dolado, Ramón López-Higes, Belinda M. Brown, Fernando Maestú

**Affiliations:** 1 Collaborative Genomics Group, School of Medical and Health Sciences, Edith Cowan University, Joondalup, Western Australia 6027 Australia; 2 Biological and Health Psychology Department, School of Psychology, Universidad Autonoma de Madrid, 28049 Madrid, Spain; 3 Laboratory of Cognitive and Computational Neuroscience (UCM-UPM), Center for Biomedical Technology, Parque Científico y Tecnológico de la UPM, Crta. M40, Km. 38, 28223 Pozuelo de Alarcón, Madrid Spain; 4 Experimental Psychology Department, School of Psychology, Universidad Complutense de Madrid, 28223 Pozuelo de Alarcon, Spain; 5 Departamento de Nutricion y Ciencia de los Alimentos, Facultad de Farmacia, Universidad Complutense de Madrid, 28040 Madrid, Spain; 6 IMDEA-Food, CEI UAM + CSIC, Madrid, 28049 Spain; 7 Department of Psychobiology and Methodology in Behavioral Sciences, Universidad Complutense de Madrid (UCM), Pozuelo de Alarcón, 28223 Spain; 8 Networking Research Center on Bioengineering, Biomaterials and Nanomedicine (CIBER-BBN), 28029 Madrid, Spain; 9 Departamento de Especialidades Medicas y Salud Pública, Universidad Rey Juan Carlos, 28922 Alcorcon, Spain; 10 Centro de Investigación Biomédica en Red de Diabetes y Enfermedades Metabólicas Asociadas, 28040 Madrid, Spain; 11 Physiotherapy and Nursing Faculty, University of Castilla-La Mancha, Toledo, 45004 Spain; 12 Endocrinology and Nutrition Department, Hospital Clinico San Carlos and Instituto de Investigación Sanitaria del Hospital Clínico San Carlos, 28040 Madrid, Spain; 13 Facultad de Psicología, Centro Universitario Villanueva, 28034 Madrid, Spain; 14 School of Pharmacy and Biomedical Sciences, Faculty of Health Sciences, Curtin Health Innovation Research Institute, Curtin University, Bentley, Western Australia 6102 Australia; 15 Neurology Department, Hospital Clinico San Carlos and Instituto de Investigación Sanitaria del Hospital Clínico San Carlos, 28040 Madrid, Spain; 16 Discipline of Exercise Science, College of Science, Health, Engineering and Education, Murdoch University, Murdoch, Western Australia 6150 Australia

**Keywords:** *APOE* ε4, Magnetoencephalography, Alzheimer’s disease, Physical activity, Alpha power

## Abstract

**Background:**

Electrophysiological studies show that reductions in power within the alpha band are associated with the Alzheimer’s disease (AD) continuum. Physical activity (PA) is a protective factor that has proved to reduce AD risk and pathological brain burden. Previous research has confirmed that exercise increases power in the alpha range. However, little is known regarding whether other non-modifiable risk factors for AD, such as increased age or *APOE* ε4 carriage, alter the association between PA and power in the alpha band.

**Methods:**

The relationship between PA and alpha band power was examined in a sample of 113 healthy adults using magnetoencephalography. Additionally, we explored whether ε4 carriage and age modulate this association. The correlations between alpha power and gray matter volumes and cognition were also investigated.

**Results:**

We detected a parieto-occipital cluster in which PA positively correlated with alpha power. The association between PA and alpha power remained following stratification of the cohort by genotype. Younger and older adults were investigated separately, and only younger adults exhibited a positive relationship between PA and alpha power. Interestingly, when four groups were created based on age (younger-older adult) and *APOE* (E3/E3-E3/E4), only younger E3/E3 (least predicted risk) and older E3/E4 (greatest predicted risk) had associations between greater alpha power and higher PA. Among older E3/E4, greater alpha power in these regions was associated with improved memory and preserved brain structure.

**Conclusion:**

PA could protect against the slowing of brain activity that characterizes the AD continuum, where it is of benefit for all individuals, especially E3/E4 older adults.

## Introduction

As life expectancy increases worldwide, we are witnessing an increase in the prevalence of age-related diseases, such as Alzheimer’s disease (AD) [61]. AD is a neurodegenerative disease of unknown etiology characterized by progressive cognitive impairment that prevents the individual from engaging in an active, independent life and ultimately causes death [24, 62]. Although the clinical onset of AD usually occurs at 70 years on average [13], strong evidence suggests that the underpinning neuropathological process begins up to 20 years before the appearance of the first symptoms [4, 9, 35, 36]. AD is a multifactorial disease that is influenced by a combination of environmental and genetic factors, where age and the carriage of the apolipoprotein E (*APOE*) ε4 allele are considered the greatest non-modifiable risk factors [19, 42].

Several neuroimaging techniques have been utilized to investigate AD disease progression. This work has identified a slowing of background brain activity throughout the AD continuum, mainly over occipital and parietal regions, as measured through magnetoencephalography (MEG) or electroencephalography (EEG) [1, 2, 49, 67]. More specifically, decreases in the peak and median frequencies of the power spectrum have been consistently described [18, 58], which is thought to be driven by both an enhancement of low frequencies (2–8 Hz) and a decrease in high frequencies (8–45 Hz) [3, 55, 59, 60]. Particularly relevant is the activity within the alpha band (8–12 Hz), which usually comprises the peak of the power spectrum. Power in the alpha band has been the focus of attention in many studies targeting different stages of preclinical and prodromal AD, as well as clinically symptomatic AD. Alterations of this kind of activity have been detected in asymptomatic ε4 carriers [25] and in individuals with subjective cognitive decline (SCD) [44] and mild cognitive impairment (MCI) [28], and correlate with cognitive worsening. Power reduction in the alpha band is thought to be the result of cumulative synaptic damage, mostly of cholinergic connections, which impedes the synchronization of activity at higher frequencies [16, 55].

While there is currently no cure for AD, several lifestyle factors have been identified to prevent or slow down disease progression. Physical activity (PA) is one of the most relevant protective factors within this field of study [38, 77]. Several studies suggest that PA triggers a series of neuroprotective mechanisms in the brain that effectively reduce AD risk and neuropathological burden [6, 17, 37, 42, 53]. Additionally, previous literature has shown that both acute and chronic PA are associated with higher peak frequencies and power in the alpha band [25, 31, 39]. Less well-known is how risk factors for AD, such as increased age and *APOE* ε4 carriage, influence this relationship. In this line, ε4 carriage has been associated with lower age at onset and has a greater impact on brain pathology at earlier disease stages [13, 75]. It is crucial to understand whether individuals who are more likely to develop AD can also benefit from a more active lifestyle. We have previously shown that highly active older adults (age 60–80) present with higher alpha peak frequencies, although this relationship only existed among ε4 non-carriers [25].

In this study, we aim to provide a detailed characterization of the relationship between PA and activity in the alpha band, and the role of age and *APOE* ε4 carriage as potential moderators of this relationship. With that purpose, we will examine MEG recordings at rest from a sample of younger (48–59 years) and older adults (60+) with available objective measures of PA, classified as *APOE* E3/E3 or E3/E4. Based on current literature, we hypothesize that PA will be associated with higher power in the alpha band in posterior brain regions. However, we believe that E3/E4 carriage could determine the time window during which PA could more effectively impact brain function, so that this relationship will be stronger in younger E3/E4, compared with older E3/E4.

## Materials and methods

### Participants

A sample of 262 individuals participated in a research project aimed to study the neurophysiological features of healthy and pathological aging, with a particular interest in the recruitment of individuals at increased risk for AD. Participants were invited to join the study from local hospitals and associations and through several dissemination talks. A team of expert neuropsychologists ensured that individuals willing to participate met inclusion criteria. The list of exclusion criteria has been detailed previously [25]. Participants were asked to provide signed informed consent. The Institutional Review Board Ethics Committee at Hospital Universitario San Carlos approved the study protocol, and the procedure was performed following the Helsinki Declaration and national and European Union regulations.

From the original cohort, we only included participants who had available and valid data regarding our main variables of interest (*n* = 191; Mini-Mental State Examination (MMSE) score, genetic information, and validated magnetic resonance imaging (MRI), MEG, and actigraphy data). Additionally, we excluded anyone aged less than 45 years (*n* = 6), with an MMSE score less than 26 (*n* = 7), or carrying less frequent *APOE* genotypes (ε2ε2, *n =* 2; ε2ε3, *n =* 14; ε2ε4, *n =* 2; ε4ε4, *n =* 5). We focused on the comparison between individuals at standard genetic risk for AD (ε3ε3) and individuals at increased genetic risk for AD in heterozygosis (ε3ε4) since sample sizes were insufficient to separately study the effects of ε2 carriage (linked to reduced risk of AD but increased risk of type III hyperlipoproteinemia [75]) and ε4 carriage in homozygosis. Nevertheless, excluded genotypes are known to alter molecular and cellular dynamics [22, 75], which could potentially interfere with the neurophysiological response to exercise, and therefore, we decided not to group together all ε4 carrying (ε2ε4, ε3ε4, ε4ε4) and all ε4 non-carrying (ε2ε2, ε2ε3, ε3ε3) genotypes. Also, participants aging less than 60 were considered young adults (45–59) and those aging 60 and above were considered old adults (60–82). Accordingly, the remaining 155 participants were then categorized into one of four groups: young E3/E4 (*n* = 30), old E3/E4 (*n* = 16), young E3/E3 (*n* = 61), and old E3/E3 (*n* = 48). To ensure all groups were matched with regard to PA levels, sex, educational level, MMSE, and body mass index, a subset of participants from each group were selected for inclusion: young E3/E4 (*n* = 20), old E3/E4 (*n* = 16), young E3/E3 (*n* = 44), and old E3/E3 (*n* = 33). Additionally, we made sure that genotype groups would match in age (E3/E3 and E3/E4) and age groups would match in the percentage of E3/E4 (younger adults and older adults). Reasons to match the sample according to all these relevant variables instead of using them as covariates in subsequent analyses have been reported previously [14].

The final sample was composed of 113 healthy adults, aged 48–82 years. A detailed list of the sample characteristics can be found in Table 1, including scores extracted from the neuropsychological tests: Geriatric Depression Scale [76], the anxiety subscale from the Goldberg Anxiety and Depression Inventory [33], and the Digit Span (an index was created using forward and backward spans) and Logical Memory II (an index was created using gist immediate and delayed recall) subscales from the Weschler Adult Intelligence Scale IV (WAIS-IV, [73]), as well as some potential confounding variables, such as education, anxiety, depression, and body mass index (BMI).

**Table 1:** Descriptive measures of the final sample

***Variable***	***Whole sample***	***YOUNG34*** (***N = 20***)	***OLD34*** (***N = 16***)	***YOUNG33*** (***N = 44***)	***OLD33*** (***N = 33***)	***Group comparison***
**Sex (M; F)**	31; 82	4; 16	3; 13	12; 32	12; 21	χ^2^(3, *N* = 113) = 2.484, *p* = 0.482
**Age**	59.92 ± 7.52	55.05 ± 2.76	66.56 ± 5.67	54.48 ± 2.9	66.91 ± 6.27	*Y*_33_ − *Y*_34_ : *t*(62) = − 0.749, *p* = 0.478 *O*_33_ − *O*_34_ : *t*(47) = 0.187, *p* = 0.853
**Education**	4.60 ± 0.62	4.55 ± 0.69	4.8 ± 0.41	4.64 ± 0.57	4.48 ± 0.71	*F*(3, 108) = 0.987, *p* = 0.402, *η*^2^ = 0.027
**MMSE**	29.16 ± 0.94	29.25 ± 0.97	29.25 ± 0.86	29.07 ± 0.87	29.18 ± 1.07	*F*(3, 109) = 0.250, *p* = 0.861, *η*^2^ = 0.007
**Depression**	1.29 ± 1.39	1.42 ± 1.64	1.27 ± 1.10	1.38 ± 1.41	1.10 ± 1.37	*F*(3, 100) = 0.288, *p* = 0.834, *η*^2^ = 0.009
**Anxiety**	1.82 ± 2.12	1.80 ± 2.19	2.11 ± 2.67	1.89 ± 2.14	1.63 ± 1.96	*F*(3, 96) = 0.140, *p* = 0.936, *η*^2^ = 0.004
**BMI**	24.98 ± 3.56	24.53 ± 2.74	24.42 ± 3.85	25.53 ± 4.16	24.76 ± 2.98	*F*(3, 107) = 0.613, *p* = 0.608, *η*^2^ = 0.017
**Physical activity**	0.012 ± 0.012	0.011 ± 0.010	0.010 ± 0.012	0.015 ± 0.013	0.011 ± 0.012	*F*(3, 108) = 1.006, *p* = 0.393, *η*^2^ = 0.027
**Total gray matter (× 10**^**3**^**)**	5.73 ± 0.51	5.80 ± 0.40	5.48 ± 0.47	5.87 ± 0.55	5.62 ± 0.48	***F***(**3**, **107**) ***=*** **3.193*, p =*** **0.027*, η***^**2**^ ***=*** **0.082** ***Y***_**33**_ **−** ***O***_**33**_ (***p*** **= 0.028**)**,** ***Y***_**33**_ **−** ***O***_**34**_ (***p*** **= 0.009**)
**Precuneus (× 10**^**3**^**)**	8.46 ± 1.04	8.62 ± 1.06	8.10 ± 0.93	8.82 ± 1.13	8.05 ± 0.75	***F***(**3,107**) ***=*** **4.706*, p =*** **0.004*, η***^**2**^ ***=*** **0.117** ***Y***_**33**_ **−** ***O***_**33**_ (***p*** **= 0.001**)**,** ***Y***_**33**_ **−** ***O***_**34**_ (***p*** **= 0.014**) ***Y***_**34**_ **−** ***O***_**33**_ (***p*** **= 0.046**)
**Hippocampus (×10**^**3**^**)**	3.71 ± 0.41	3.79 ± 0.39	3.44 ± 0.38	3.83 ± 0.38	3.63 ± 0.40	***F***(**3,107**) ***=*** **4.610*, p =*** **0.004*, η***^**2**^ ***=*** **0.114** ***Y***_**33**_ **−** ***O***_**33**_ (***p*** **= 0.031**)**,** ***Y***_**33**_ **−** ***O***_**34**_ (***p*** **= 0.001**) ***Y***_**34**_ **−** ***O***_**33**_ (***p*** **= 0.009**)
**Episodic memory**	24.28 ± 5.13	24.35 ± 4.97	23.09 ± 6.32	24.05 ± 4.78	25.16 ± 5.49	*F*(3, 96) = 0.466, *p* = 0.707, *η*^2^ = 0.014
**Working memory**	10.26 ± 2.10	10.37 ± 2.19	10.00 ± 2.31	10.20 ± 1.95	10.39 ± 2.21	*F*(3, 108) = 0.151, *p* = 0.929, *η*^2^ = 0.004

Mean values ± standard deviation were provided for sample characteristics as well as variables used for correlation analyses. These include the following: sex (where M stands for male and F for female); age (in years); education (in terms of educational level on a 0—illiterate—to 5—postsecondary education—scale); Mini-Mental State Examination (MMSE); anxiety (Goldberg’s test); depression (Geriatric Depression Scale); body mass index (BMI); total physical activity (TPA, normalized by actigraphy wear time); total gray matter (GM), hippocampal, and precuneal volumes (bilateral average, in mm^3^); episodic memory (Logical Memory II Index: immediate and delayed recall for gist); and working memory (Digit Span Index: direct and reverse). Results are displayed for the whole sample and also for each subsample of interest (young E3/E4—Y34; old E3/E4—O34; young E3/E3—Y33; and old E3/E3—O33). Chi-squared test was calculated for sex, Student’s *t* test to compare within age groups, and ANOVAs for the rest of the variables. When significant differences between groups were found, least significant difference post hoc measures were calculated and significant *p* values are shown and marked in bold. No significant between-group differences arose across most comparisons, except for GM volumes, where older groups presented smaller volumes

### Physical activity measurement

An ActiGraph GT3X+ accelerometer (LLC, Pensacola, FL) was provided to every participant, and they were requested to wear the device on their right hip for 7 complete days. They were advised to only take the ActiGraph off during water-based activities [11, 12]. ActiLife software (6.13.3) (LLC, Pensacola, FL) was used to clean and process the acquired data. To meet validation criteria, each individual should have worn the accelerometer for a minimum of 10 h per day during at least 3 weekdays and 1 weekend day [11]. Non-wear time was defined as ≥ 60 min of continuous zeroes, allowing for up to 2 min of ≤ 100 counts [69]. For this study, we considered a standardized measure of total PA (TPA) volumes calculated by ActiLife: Total Time In Freedson Bouts. TPA was normalized by total wear time.

### APOE genotyping

Genomic DNA was extracted from 10 ml blood samples in ethylenediaminetetraacetic acid (EDTA). TaqMan assays were used on an Applied Biosystems 7500 Fast Real Time PCR machine (Applied Biosystems, Foster City, CA) to determine single nucleotide polymorphisms (SNPs) rs7412 and rs429358 genotypes and establish *APOE* haplotypes accordingly. As mentioned above, only ε3ε3 and ε3ε4 individuals were included in this study.

### MRI acquisition and volumetric analyses

T1-weighted MRI images from each participant were generated using a General Electric 1.5 T system and applying a high-resolution antenna and a homogenization PURE filter (Fast Spoiled Gradient Echo sequence, TR/TE/TI = 11.2/4.2/450 ms; flip angle 12°; 1 mm slice thickness, 256 × 256 matrix, and FOV 25 cm). Freesurfer software (version 6.1.0) was used for automated cortical parcellation and subcortical segmentation [21]. The measures that were included in further analyses were total gray matter (GM), precuneal, and hippocampal volumes (in mm^3^). The volumes of bilateral structures were collapsed in order to obtain a single measure for each region.

### Magnetoencephalography

Neurophysiological data was acquired using a whole-head Elekta-Neuromag MEG system with 306 channels (Elekta AB, Stockholm, Sweden) at the Center for Biomedical Technology (Madrid, Spain). MEG data was collected at a sampling frequency of 1000 Hz and online band-pass filtered between 0.1 and 330 Hz.

All subjects underwent a 5-min eyes-closed resting-state MEG recording while sitting comfortably inside of a magnetically shielded room. They were requested to stay awake and to minimize their body movements. Each subject’s head shape was defined relative to three anatomical locations (nasion and bilateral preauricular points) using a 3D digitizer (Fastrak, Polhemus, VT, USA), and head motion was tracked through four head-position indicator (HPI) coils attached to the scalp. These HPI coils continuously monitored the subjects’ head movements, while eye movements were monitored by a vertical electrooculogram assembly (EOG) composed of a pair of bipolar electrodes. Raw recording data was first introduced to Maxfilter software (v 2.2, correlation threshold = 0.9, time window = 10 s) to remove external noise using the temporal extension of the signal space separation method with movement compensation [68]. Then, magnetometer data [27] was automatically examined to detect ocular, muscle, and jump artifacts using Fieldtrip software [54], which were visually confirmed by an MEG expert. The remaining artifact-free data was sectioned into 4-s segments. Afterwards, independent component analysis (ICA)-based procedure was applied to remove heart magnetic field artifacts and EOG components. Only those recordings with at least 20 clean segments (80 s of brain activity) were utilized in subsequent analyses.

MEG clean time series were band-pass filtered (2 s padding) between 2 and 45 Hz. Source reconstruction was carried out using a regular grid of 1 cm spacing in the Montreal Neurological Institute (MNI) template. The resulting model comprised 2459 sources homogeneously distributed across the brain. This model was linearly transformed to each subject’s space. The leadfield was calculated using a single shell model [52]. Sources’ time series were reconstructed using a linearly constrained minimum variance beamformer [71]. Power spectrum of each grid’s node was computed by means of fast Fourier transform using Hanning tapers with 0.25 Hz smoothing. For each node, relative power was calculated by normalizing by total power over the 1.5- to 45-Hz range. Since power in the alpha band was the focus of the study, only frequency steps (25 in total) within the interval [8–14 Hz] were considered for the analyses. The source template with 2459 nodes in a 10-mm spacing grid was segmented into 78 regions of the Automated Anatomical Labeling atlas (AAL, [70]), excluding the cerebellum, basal ganglia, thalamus, and olfactory cortices. Those 78 regions of interest included 1202 of the original 2459 nodes. Trials were averaged across subjects ending up with a source-reconstructed power matrix of 1202 nodes × 25 frequency steps × 113 participants. This final power matrix was employed in the correlation analysis (see below).

### Statistical analyses

The aim of this study was the detection of any robust correlation between power values derived from clusters of nodes localized in certain brain regions and TPA. Such analysis relied on network-based statistics (NBS) [23, 78]. Clusters were built according to a criterion of spatial and frequency adjacency. Each cluster consisted of several adjacent nodes, which systematically showed a significant partial correlation (with age as covariate) in at least 3 consecutive frequency steps (a 0.75-Hz interval) between their corresponding power values and TPA (Spearman correlation *p* value < 0.01). Importantly, all nodes within a cluster must have shown the same sign of the correlation coefficient, thus indicating that the cluster might be deemed as a functional unit. Only clusters involving at least 1% of the nodes (i.e., a minimum of 12 nodes) in each frequency step were considered. Cluster-mass statistics were assessed through the sum of the Spearman rho values across all nodes and significant frequency steps.

Then, to control for multiple comparisons, the entire analysis pipeline was repeated 5000 times, shuffling the correspondence between power estimates and TPA across subjects. At each repetition, the maximum statistic of the surrogate clusters (in absolute value) was kept creating a maximal null distribution that would ensure control of the family-wise error rate (FWER) at the cluster level. Cluster-mass statistics on each cluster in the original dataset were compared with the same measure in the randomized data. The NBS *p* value represented the proportion of the permutation distribution with cluster-mass statistic values greater or equal than the cluster-mass statistic value of the original data.

Power values were averaged across all nodes and frequencies that belonged to the cluster. Such average was considered as the representative power marker value for that cluster, and further subjected to new correlation analyses. Therefore, the statistics presented in the “Results” section derived from the correlation between the averaged power value of each significant cluster and the corresponding TPA for each participant. As it has been mentioned above, correlations were first performed within the whole sample. In a second step, correlations between power and TPA scores were performed independently for all subgroups within the sample (E3/E3, E3/E4, younger adults, older adults, young E3/E3, young E3/E4, old E3/E3, and old E3/E4). Additionally, moderation analyses were carried out to study the possible influence exerted by either age (younger vs older adults) or *APOE* (E3/E4 vs E3/E3) in the reported relationship between average power and TPA. To this aim, we employed multiple regression analysis and calculated the increase in variance explained by our model after including the interaction term in two separate models (i.e., APOE*TPA and age*TPA, model_1 and model_2 respectively). These models used TPA and *APOE* (model_1)/age (model_2) as predictors to linearly estimate average power in the significant cluster. In a second step, the interaction term for each specific model was added (TPA*APOE or TPA*age). The *p* value for this interaction term is interpreted as the moderating effect significance.

Finally, average power values were correlated with measures of memory performance (working an episodic memory) and GM volumes (total gray matter, precuneus, and hippocampus), traits that are known to be affected early in AD. These analyses were only carried out for those subgroups showing a significant correlation between TPA and average power (the complete list is shown in Table 2). Statistical analyses were carried out using Matlab R2017a (Mathworks Inc).

**Table 2:** AAL ROIs that were partially captured by the significant cluster

***ROI***	***Percentage of ROI occupied***
Right precentral gyrus	88.89
Right postcentral gyrus	63.64
Left precuneus	67.86
Left middle occipital lobe	58.62
Right superior parietal gyrus	83.33
Left superior parietal gyrus	81.25
Right angular gyrus	72.22
Left postcentral gyrus	35.29
Right inferior parietal gyrus	100.00
Right superior frontal gyrus	25.81
Right precuneus	38.10
Right inferior frontal gyrus, opercular	46.15
Left superior occipital lobe	54.55
Right cingulate gyrus, middle part	33.33
Left cuneus	45.45
Right supramarginal gyrus	50.00

Listed are the regions of interest (ROIs) from the Automated Anatomical Labeling (AAL) atlas that are part of the significant cluster where physical activity correlates with power in the alpha band. It shows as well the percentage of each ROI that is captured by that cluster

## Results

**Fig. 1:**
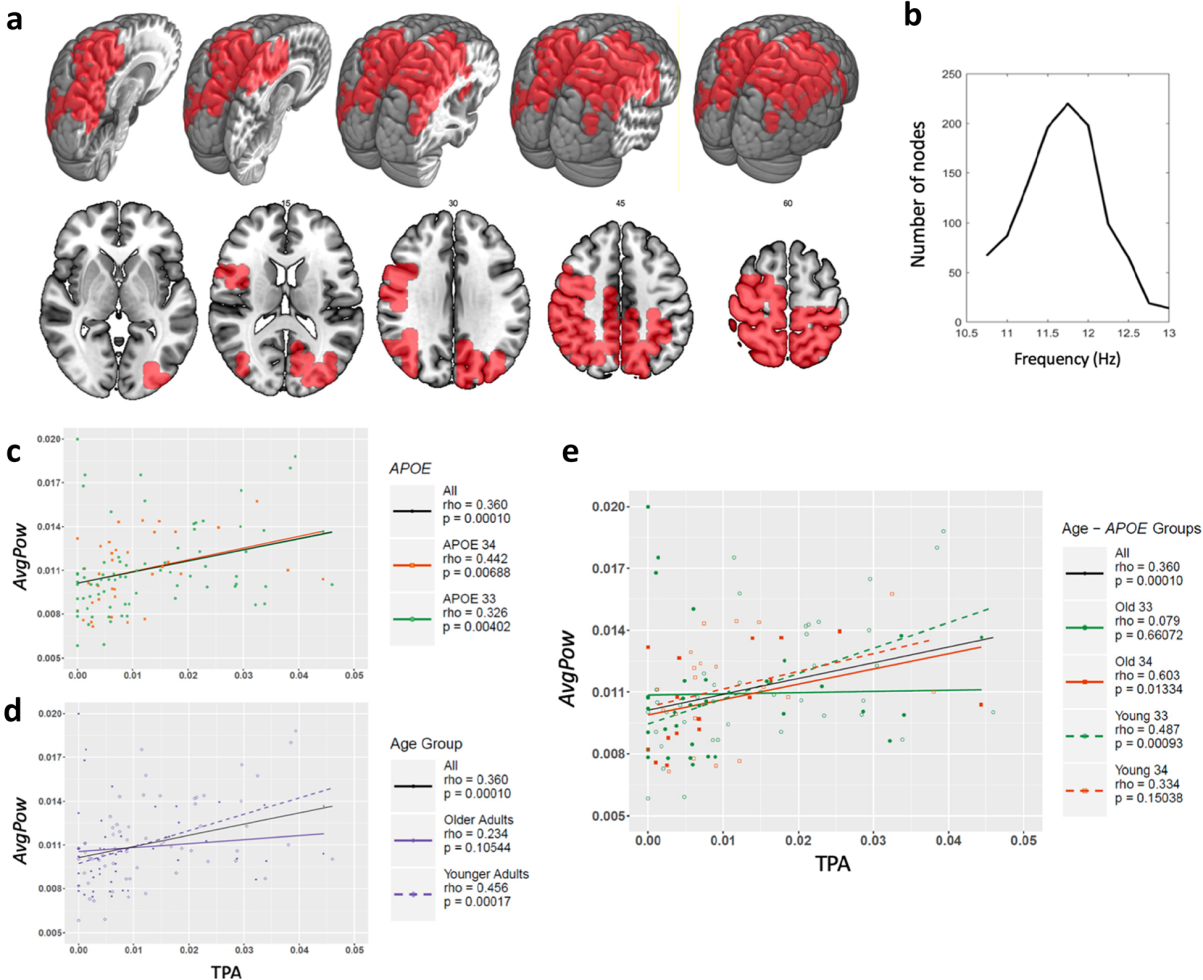
Significant cluster at 10.75–13 Hz. The brain regions comprised within the significant cluster (depicted in red; **a**), the evolution of the cluster size through the different frequency steps (**b**; maximum size at 11.75 Hz), and the scatter plots showing the correlation between the cluster’s average power (AvgPow) and total physical activity (TPA) and each genotype subgroup of the sample (*APOE* ε4 carriers—APOE 34—and non-carriers—APOE 33; **c**), each age group (younger and older adults; **d**), and all four subgroups (**e**)

A significant cluster was found in the frequency interval [10.75–13 Hz] mainly comprising posterior regions of the brain (see Fig. 1a and Table 2). The power in all frequencies of this interval positively correlated with TPA across the whole sample (rho = 0.360, *p* < 0.0001). The maximum cluster size was found at 11.75 Hz (220 nodes). The cluster size oscillates between a minimum of 67 nodes at the beginning of the frequency range and 14 at the end of that frequency range (see Fig. 1b). 11.75 Hz showed the highest average correlation coefficient value across all nodes of the cluster; rho = 0.3029.

The correlation between TPA and power in the [10.75–13 Hz] interval within the cluster generated in the previous step remained significant for both genotype groups (E3/E3: rho = 0.326, *p* = 0.004; E3/E4: rho = 0.442, *p* = 0.007; see Fig. 1c) and for younger adults (rho = 0.456, *p* < 0.001), although not for older adults (rho = 0.234, *p* = 0.105; Fig. 1d). In this line, only the moderator effect of age, but not that of E3/E4, was significant (respectively, *p* = 0.030 and *p* = 0.923). When both factors were simultaneously considered, only young E3/E3 (rho = 0.487, *p* < 0.001) and old E3/E4 (rho = 0.603, *p* = 0.013) presented a significant correlation (Fig. 1e). Importantly, there were no significant differences in power in the [10.75–13 Hz] range among the four subgroups.

The average power value of the significant cluster was used for new correlation analyses in younger E3/E3 and older E3/E4. Alpha power in posterior brain regions correlated with preserved brain structure and improved working and episodic memory, but only among older E3/E4. The complete set of correlations is displayed in Table 3.

**Table 3:** Average power correlations

Variable	OLD34			YOUNG33		
	***N***	Rho	***p*** value	***n***	Rho	***p*** value
**Total gray matter**	16	0.624	0.012*	43	− 0.017	0.915
**Precuneus**	16	0.515	0.044*	42	0.117	0.460
**Hippocampus**	16	0.271	0.310	43	− 0.081	0.605
**Episodic memory**	11	0.881	< 0.001*	43	0.181	0.246
**Working memory**	16	0.587	0.017*	43	− 0.220	0.156

Results for the Spearman correlation analyses between average power in the significant cluster and different measures of structural integrity and memory performance, including total gray matter (GM), hippocampus, and precuneus volumes (bilateral average, in mm3); episodic memory (Logical Memory II Index: immediate and delayed recall for gist); and working memory (Digit Span Index: direct and reverse). Correlation analyses were carried out for the two subgroups that showed a positive relationship between physical activity and alpha power in the significant cluster: old E3/E4—OLD34—and young E3/E3—YOUNG33. P values lower than 0,05 are indicated with an asterisk and marked in bold. Only old E3/E4 presented an association between alpha power and cognitive performance/GM volumes

## Discussion

The slowing of background brain activity, usually defined as a shift to the left in the power spectrum, is a consolidated marker of aging and brain disease which has been used to monitor pathological progression along the AD continuum. In this study, we aimed to characterize the relationship between PA and activity in the alpha band, and the role of age and *APOE* ε4 carriage as potential moderators of this relationship. In a previous publication, we reported that greater self-reported PA was significantly associated with greater alpha peak frequencies in older adults. Moreover, we were able to observe that such relationship remained significant only among E3/E3, although the small sample size in the E3/E4 group did not allow us to reach a definite conclusion [25]. In this study, taking a different approach, we have been able to detect a positive relationship between objectively measured PA (in terms of total volume) and power in the alpha band in a cluster comprising mainly occipital and parietal regions, which are classic sources of this brain rhythm. More importantly, this effect persisted in both E3/E4 and E3/E3. However, when we split the sample into younger and older adults, only younger adults exhibited greater alpha power at greater PA volumes. Interestingly, when we further divided the sample considering both risk factors combined (age and genotype), only young E3/E3 and old E3/E4 showed the beneficial association with PA.

Late onset Alzheimer’s disease is a multifactorial disease, which means that different risk and protective, environmental, and genetic factors interact throughout the lifespan to determine the likelihood that an individual will develop the disease. PA has been well-established as an important neuroprotective element that has the potential to decrease AD risk and improve AD-related neuropathological burden [6, 17, 53]. In fact, both greater power in the alpha band and a more active lifestyle have been reported to be associated to an individual’s capacity to generate long-term potentiation-like synaptic plasticity [65]. PA presents the advantage of being a modifiable lifestyle variable. Individuals can purposefully increase their PA levels in order to improve their health outcomes. On the other side, age and *APOE* ε4 carriage are considered the two non-modifiable risk factors for AD that have the greatest impact on the probability that an individual will develop AD [19, 42]. For this reason, it is important to understand how these elements interplay to shape brain structure and function.

As it has already been mentioned, increasing age is associated with greater risk for AD and lower power in the alpha band at rest. Here, we report that the potentially protective association between PA and greater alpha power is stronger in younger adults (48–59 years). This finding does not necessarily contradict the previous finding showing that the alpha peak frequency at rest was higher in highly active older adults. Both features (alpha peak and alpha power) are closely related, and they are affected by the same processes that cause the loss of (mainly cholinergic) synapses [16, 51, 55]. Still, they reflect distinct aspects of the same phenomenon, which could be differentially modulated by various external factors along the disease continuum. Power is highly influenced by the number of simultaneously active synapses. Even in the absence of brain disease, aging is characterized by a reduction in power in high frequencies [30]. Therefore, PA might not be able to increase power as effectively in older adults, due to neuronal damage inherent to aging. However, it seems to still be able to exert a positive effect, as reflected by a positive association with the peak frequency. In fact, the power frequency range we found to be associated with PA levels (10.75–13 Hz) does not overlap with (it is higher than) the average alpha peak previously reported for healthy adults [26].

Other studies, however, have reported that acute PA (e.g., a single bout of aerobic exercise) has a greater effect on spectral measures [15] and cognitive outcomes [8] in older adults, although Moraes et al. [48] only found significant effects in young adults. The acute neurophysiological response to PA, especially the enhancement of theta activity, is believed to serve a cognitive function to overcome exercise-related cognitive challenges [34]. In these studies, older individuals presented lower baseline power across different frequency ranges (alpha, theta, and delta) than younger individuals. The increase in power produced by acute PA, which immediately reverts after exercise ceases [40, 41], was greater in older individuals, while still not reaching the power levels featured by younger participants. Therefore, there seems to be more room for improvement among older adults, which is promoted by acute PA. However, habitual engagement in PA could possibly have greater potential to constrain the slowing of background (resting) activity among younger individuals. Thus, it is reasonable to suggest that the immediate and long-term beneficial effects of PA in the brain could be mediated by different mechanisms which might not be equally prominent at different life stages. For example, different neurotransmitters could be responsible for such diverse effects, which in turn could possibly be differentially altered in aging and disease [46, 48].

Alternatively, it is possible that with aging, there is a shift from posterior to anterior background activity sources. In fact, a few studies showing that alpha power could increase in older adults with acute, habitual physical exercise or after an exercise-based intervention focused on frontal activity sources [10, 64, 72]. Here, when we studied the effect of age, we focused on a cluster comprising parieto-occipital brain regions where the relationship between PA and alpha power was maximized across the whole sample. Hence, it remains possible that older adults present a weaker association limited to the brain sources explored here. It is also important to bear in mind that in previous studies, both the intensity and the duration of acute exercise have been found to influence the specific neural response evoked [47, 74], which could also explain certain differences across studies.

*APOE* ε4 carriage poses the greatest genetic risk factor for AD. There is controversy in the field regarding whether ε4 carriers and non-carriers benefit to the same extent from PA engagement. Many studies conclude that physical inactivity is particularly harmful for ε4 carriers [5, 32, 45, 63, 66]. Yet, a considerable number of publications fail to find benefits among ε4 carriers, suggesting that the presence of this allele could impair the mechanisms through which PA exerts its action [7, 20, 56]. Nevertheless, these apparently conflicting results are a consequence of the employment of different study designs to evaluate a wide variety of outcomes. In the present study, we were able to replicate the association between PA and alpha power described for the sample as a whole when looking separately at E3/E4 and E3/E3. Although ε4 carriage can worsen cholinergic deficits [57], which could explain previously reported alterations in alpha activity, PA is equally associated with higher alpha power regardless of genetic burden.

Here, we describe for the first time that only young E3/E3 and old E3/E4 present a significant positive relationship between PA and alpha power. Such findings contradict our original hypothesis that, in E3/E4, PA would be associated with better outcomes at earlier stages in life. We would have thought that the presence of this risk allele would accelerate brain aging and pathology, hence bringing forward the time window during which PA could perform its protective role. In this line, in AD patients, age and *APOE* interactions had been previously described, where cerebral glucose metabolism disruption was steeper among carriers through the aging process [50]. It seems though that PA in midlife is associated with greater alpha power only among ε4 E3/E3. One possible explanation is that the benefits of PA are maximal in individuals at opposite ends of the risk spectrum. On the one end, young individuals who do not present increased genetic risk for AD are able to generate a positive response to exercise, since the machinery that mediates these effects is in optimal conditions. On the other end, old individuals at increased genetic risk are at a more vulnerable position where even the slightest changes could result in meaningful improvements. This is coherent with the idea that only in this group power correlated with improved memory and better-preserved brain structure. In other words, local increases in alpha power in older ε4 E3/E4 could serve a compensatory purpose. However, mean age in our sample, even in the older groups, was still below previously reported mean age at disease onset [13]. Therefore, it is possible that the beneficial effects that E3/E4s show in their 60s could be observable in E3/E3 at later stages that were not covered within our age range (they might not need to activate compensation mechanisms yet), when pathological burden among E3/E4 might be too advanced to allow circuit reorganization. More research is needed to fully elucidate the processes that give rise to these seeming interactions between PA, age, and *APOE* genotype. In particular, it would be interesting to increase the participants’ age range in future studies. Including individuals in their 80s and 90s (when greater damage is expected due to the aging process and also the risk of AD is higher) would enable us to test whether older E3/E3 show a significant relationship between PA and power in the alpha band within the identified cluster. Also, by including younger individuals in their 20s and 30s, we could explore whether young E3/E4 show this effect before the detrimental effects of *APOE* ε4 are more notorious. Additionally, we plan to follow up this cohort every 3 years. Longitudinal changes within this same sample will provide highly valuable information to understand the underpinnings of the effects here reported.

The present study combines a series of strengths that confer it a novel perspective. Accelerometers were used to obtain objective estimates of PA, which were later used as continuous measures, thus avoiding the loss of statistical power that comes with the stratification in different PA levels. In this line, over-recruitment of E3/E4 enabled the careful examination of the interplay among a series of modifiable and non-modifiable factors that impact dementia risk. Age and genetic risk for AD were considered separately and, for the first time, jointly as potential limiting factors that could constrain the relationship between PA and synaptic function. In addition, we used a completely data-driven methodology that enables the automatic delimitation of the spatial and frequential dimensions of the clusters. Finally, brain activity data was collected through MEG, which offers ample temporal and spatial resolutions [43]. Remarkably, this technique enables the measurement of synaptic activity, which is early affected in the AD continuum and, as we have discussed here, can be enhanced through PA. Neurophysiological approaches (i.e., MEG and EEG) in the study of AD characterization and prevention present the advantage of being completely non-invasive techniques that can easily be repeated over time. Therefore, they offer great potential to be used in the process of monitoring disease progression or assessing the impact of different kinds of interventions.

## Limitations

Overall, this study endeavors to deepen our understanding of how PA could serve a protective role in the prevention of AD, even in the presence of non-modifiable risk factors. Still, numerous questions remain unanswered which future studies should address. For example, the cross-sectional nature of this study does not enable the establishment of a causal relationship between PA and brain activity. Also, we did not include ε4ε4 homozygotes, due to the small sample size of this group in our cohort, and therefore, we could not explore dosage effects. In this line, a greater sample size would have enabled us to perform a three-way interaction analysis to properly asses the combined moderating effect of *APOE* and age on PA. Finally, although objective measures of PA suppose an improvement with respect to self-reported PA in terms of precision, they do not reflect individual levels of exertion [29], which are more closely related to the physiological response to exercise [37].

## Conclusions

The current study provides new insights and builds on previous literature on the *APOE* ε4 modulation of the neural effects of PA. In particular, this study provides evidence that age should be considered in the attempt to disentangle seemingly mixed results from earlier investigations. Our results show, for the first time, that only young E3/E3 and old E3/E4 carriers present a significant positive relationship between PA and alpha power and that, in the latter group, such higher alpha power is also associated with better memory performance and GM preservation. Above all, this study highlights the importance of implementing public policies that promote physical activity in midlife and late life.

## Data Availability

The datasets used and/or analyzed during the current study are available from the corresponding author on reasonable request.

## Abbreviations

AAL: Automated Anatomical Labeling
AD: Alzheimer’s disease
APOE: Apolipoprotein E
AvgPow: Average power
BMI: Body mass index
EDTA: Ethylenediaminetetraacetic acid
EEG: Electroencephalography
EOG: Electrooculogram
F: Female
HPI: Head-position indicator
GM: Gray matter
M: Male
MCI: Mild cognitive impairment
MEG: Magnetoencephalography
MMSE: Mini-Mental State Examination
MNI: Montreal Neurological Institute
MRI: Magnetic resonance imaging
NBS: Network-based statistics
PA: Physical activity
ROI: Region of interest
SCD: Subjective cognitive decline
SNPs: Single nucleotide polymorphisms
TPA: Total physical activity
WAIS-IV: Weschler Adult Intelligence Scale IV
